# Evaluating Cognitive Load in Clinical Workflows Highlights Leverage Points for Guideline-Concordant Statin Initiation

**DOI:** 10.21203/rs.3.rs-7632374/v1

**Published:** 2025-10-27

**Authors:** Ratnalekha V. N. Viswanadham, Yuhan Cui, Priyanka Solanki, Nicole Redfern, Amelia Shunk, Angela Mastrianni, Defne L. Levine, Devin M. Mann, Safiya I. Richardson

**Affiliations:** NYU Grossman School of Medicine; NYU Grossman School of Medicine; NYU Langone Health; NYU Grossman School of Medicine; Tulane University School of Medicine; NYU Grossman School of Medicine; NYU Grossman School of Medicine; NYU Grossman School of Medicine; NYU Grossman School of Medicine

**Keywords:** statin, clinical decision support, EHR, process mining, cognitive load, audit log data, machine learning

## Abstract

**Introduction:**

Linking EHR use to care quality offers insights for interventions to improve guideline adherence and close care gaps. We examine how EHR metadata can measure cognitive load in primary care providers during statin prescribing and identify points of cognitive load in the EHR workflow.

**Methods:**

EHR primary care encounter data from a large academic health system in 2024 were retrospectively extracted. We identified adult patients who met the criteria for statin initiation and calculated their ASCVD risk scores. Cognitive load metrics were derived from EHR metadata. Logistic regressions evaluate associations between cognitive load and statin initiation, adjusting for patient covariates and provider fixed effects. Gradient-boosted forests and SHAP values identified key EHR events and cognitive load associated with the initiation of statin therapy.

**Results:**

Longer encounter duration increased the likelihood of statin initiation, whereas more time spent per EHR event decreased it. Non-linear effects were observed for loop count and distinct event count: the probability of initiation decreased with increasing loop counts up to 93.9 loops, then increased beyond this threshold. For distinct events, the initiation probability increased up to approximately 18 events and declined at higher counts. In a gradient-boosted decision tree model, average time per event was the strongest predictor (72.2% relative contribution). Additional positive predictors included the time spent reviewing lab results and on suggested medication order sets. Modifying the order list and looping back to it were negatively associated with statin initiation.

**Discussion:**

EHR metadata can associate cognitive load with appropriate clinical behavior, finding nonlinear relations between cognitive load and statin initiation rates. This work highlights the need to optimize EHR systems to reduce cognitive burden and support clinical decision-making. Connecting cognitive load to prescribing behavior gives insight into how workflow adjustments and enhanced decision support can improve adherence to guidelines and patient care.

## 1 Introduction

### 1.0 Research Goals and Hypotheses

The objectives of this study were to assess how electronic health record (EHR) metadata can be used to measure cognitive load within primary care workflows related to initiating guideline-concordant statin therapy. We pursued two aims. First, we evaluated whether cognitive load metrics were associated with the likelihood of appropriate statin initiation. Second, we explored whether specific EHR workflow events could be linked to cognitive load patterns that predict statin initiation. We hypothesized that providers adhering to statin guidelines would exhibit higher cognitive effort and distinct EHR interaction patterns compared to those who did not. We expected this increased cognitive load to manifest as: (a) repeated actions to gather necessary information, (b) longer time spent per EHR activity, (c) more overall actions within the EHR, and (d) extended encounter duration. The second aim was exploratory, focusing on developing methods to connect EHR event–level cognitive load metrics with statin initiation behavior.

### 1.1 Literature contribution (Table 1)

**Table 1:** Literature Contribution Table

Problem or Issue	How can electronic health record (EHR) metadata identify ways to close clinical care gaps? What are EHR workflow cognitive load differences between providers who give appropriate care and providers who do not?
What is Already Known	Statins reduce cardiovascular risk but are under prescribed despite guidelines. Clinical decision support systems aim to improve prescribing but have low adoption. Cognitive load in providers can lead to errors and decision delays. EHR metadata captures clinician behavior and cognitive load, but few studies link it to specific clinical actions.
What this Paper Adds	A use case to associate EHR metadata metrics with care quality in healthcare.
Who would benefit from the new knowledge in this paper	Healthcare systems aiming to leverage EHR metadata to improve healthcare delivery. Health informaticians interested in leveraging data-driven approaches to improve clinical workflows. Behavioral scientists interested in understanding process workflows and sources of cognitive load in workflows. Policymakers improving quality of care through targeted interventions in EHR design and clinical guidelines.

## 2 Related Work

### 2.1 Clinical Context: Statin Prescribing for ASCVD Risk Prevention

Cardiovascular disease remains the leading cause of mortality globally, and failure to appropriately prescribe statins represents a missed opportunity for primary prevention at a population level.[1] Statins reduce the risk of major adverse cardiovascular events and mortality.[2] However, primary care providers fail to prescribe statin therapy for about half of patients meeting guideline criteria for initiation.[3–8] Among patients prescribed a statin, about two-thirds receive a lower-than-optimal dose.[5] To ensure patients are appropriately prescribed statins, providers must recognize that a patient meets one of four indications, often requiring calculating a patient’s risk of atherosclerotic cardiovascular disease (ASCVD) and prescribing a guideline-approved statin at the correct dose. Individualized risk assessment requires collecting elements from the patient’s medical history, vital signs, and laboratory results, and then using a risk calculator. The EHR presents opportunities to develop clinical decision support systems (CDSSs) that support the recognition, assessment, and management of ASCVD risk, like alerts and pre-populated medication orders. However, generally low provider adoption has limited the clinical impact of current CDSSs designed to improve guideline-concordant statin prescribing.[9–13] Additionally, evolving guidelines and varying thresholds for treatment based on age and comorbidities further complicate the prescribing process, especially without automated support.

### 2.2 EHR Metadata to Evaluate EHR Utilization

Cognitive load can influence the quality of healthcare decision-making by increasing the likelihood of delayed action, reliance on cognitive shortcuts, and misalignment with evidence-based care.[14, 15] In the EHR environment, poorly integrated workflows and excessive demands on attention have been associated with clinician burnout and suboptimal patient outcomes.[16] Quantifying cognitive load in routine practice is therefore critical for understanding its role in decision-making and identifying opportunities for improvement.[17] Traditional approaches to studying clinician-EHR interactions, such as time and motion studies, direct observation, surveys, and interviews, are resource-intensive and prone to biases like the Hawthorne Effect, where awareness of being observed alters behavior.[18] These methods may also fail to capture subtle but important real-time decision points.

EHR metadata is an automated tracking feature that logs behavior within the system (such as adding, deleting, or querying information) along with contextual information (date/time, user, and patient). EHR certification mandates this tracking for security and privacy purposes. As of 2017, 96 percent of hospitals and 80 percent of private practices that used EHRs were compliant with these criteria.[19] Specifically, EHR audit log metadata, collected for HIPAA compliance to track what providers view and click in the EHR, can serve as a non-invasive method to observe how providers and patients interact within the EHR without imposing additional burdens on clinicians.[20] For instance, audit log data can be used to measure how many times a provider clicks between the lab results and medication order screen during a visit, offering a proxy for task-switching or uncertainty. Using EHR metadata provides an analytical approach that not only captures natural interaction patterns but also enables scalable and objective measurement of cognitive load and its link to prescribing behavior, laying a foundation for future interventions that can be assessed through real-world practice patterns.

EHR audit log metadata offers expanding opportunities for health services research. [21] The clinical informatics literature has identified three metadata metrics that directly capture EHR user behavior related to measuring cognitive load: counts of actions captured by metadata, activity durations, and activity sequences.[22] However, extracting and interpreting these metrics to meaningfully reflect cognitive load is technically challenging. Raw EHR audit log metadata are high-volume, inconsistently structured across systems, and not collected with research in mind, making it challenging to align events with specific clinical decision-making moments. While the literature is limited in linking these metrics to specific healthcare research questions and in measuring the effectiveness of healthcare delivery, [23] process mining can bridge EHR metadata to desired behaviors and reveal system changes that may drive behavior change. By analyzing how these metrics relate to guideline adherence, health systems can identify workflow inefficiencies or decision points where additional support may be warranted.

This study aims to fill a critical gap by evaluating whether cognitive load, as measured through EHR metadata, differs between providers who prescribe statins to eligible patients and those who do not—an insight that could inform workflow-based interventions to close persistent care gaps in ASCVD prevention. Additionally, we aim to determine which EHR activities (e.g., medication orders, patient communications, and the use of pre-existing tools) are associated with a higher cognitive load in encounters that result in guideline-concordant statin prescribing.

## 3 Methods

We conducted a retrospective, cross-sectional, observational study using EHR audit log metadata and clinical encounter data to evaluate the association between cognitive load during primary care visits and initiation of statin therapy. This study was designed and reported in accordance with the Strengthening the Reporting of Observational Studies in Epidemiology (STROBE) guidelines.[24] This study was approved by the NYULH Institutional Review Board (IRB #i23–01349), which granted a waiver of authorization.

### 3.1 Population and Number of Observations

Data were retrospectively pulled from the EHR of a large academic health system that uses Epic Systems between January 1, 2024, and December 31, 2024, for primary care encounters of patients who qualified for statin initiation during their clinic visit.

### 3.2 Data Inclusion and Exclusion

To determine statin eligibility of adult patients between 20 and 75 years of age, we extracted laboratory data, including their most recent lipid panels and hemoglobin A1c, age, self-identified race and ethnicity, sex, smoking history, latest collected systolic blood pressure, medication history, and any previous history of ASCVD events to calculate each patient’s cardiovascular risk based on the 2013 American College of Cardiology/American Heart Association (ACC/AHA) ASCVD 10-year risk score [25] We identified each eligible patient’s most recent primary care visit in 2024 and retrieved encounter-level metadata, including provider identifiers.

We included adult patients with at least one documented primary care encounter in 2024 who met clinical guidelines for statin therapy. [26] Patients were excluded if they lacked sufficient data to calculate a valid risk score, had no primary care encounters during the study period, indicated an allergy or contraindication to a statin, or were pregnant in 2024. Patients who qualified for statin therapy were identified based on the guidelines in Table 2, which are based on the ACC/AHA Task Force on Clinical Practice’s guidelines on the management of blood cholesterol. [27] Patients were then classified based on their statin prescribing status at their latest visit: initiated, defined as those with a statin medication order documented during the encounter, and not prescribed, defined as those without a corresponding statin order despite meeting guideline-based indications. Statins initiated in 2024 can be found in Table A.1.

### 3.3 Data and Data Structure

EHR audit log metadata and relevant patient clinical information were extracted from the healthcare system’s data lake, which was built on a Hadoop distributed file system, and the relevant data were queried using MySQL databases. For patients initiated on statins in 2024, we extracted metadata from the encounter linked to the initiation. For those not initiated, we used metadata from their most recent annual wellness visit, which occurred in 2024. We defined an encounter as a unique interaction between a statin-prescribing provider (physician, physician assistant [PA], nurse practitioner [NP]) and a patient. An encounter may consist of multiple, non-contiguous time segments during which the provider engages with the patient’s data or performs related actions. For example, a provider might review the patient’s chart regarding an encounter from 1:00 PM to 1:03 PM, conduct a face-to-face visit from 1:40 PM to 2:10 PM, and later complete documentation after work hours (between 7:00 PM and 7:00 AM) or even the next day. These time segments are combined into a single encounter to reflect the total effort involved with the encounter. From the encounter’s metadata, we derived four cognitive load metrics based on the user interactions (an “event”) logged by the EHR. Events in the EHRs include instances when a provider accesses patient data, their actions related to the data, and other workflow activities associated with an ID tied to the patient encounter. The sequence of timestamped events enables the creation of metrics that represent the effort exerted by the provider when interacting with the EHR, which we refer to as cognitive load metrics. Each of these metrics was chosen because a stakeholder can introduce changes in the EHR based on a desire to alter these metrics, which would, in turn, affect cognitive load and lead to meaningful changes in clinical behavior. These metrics are elaborated on in Table 3.

### 3.4 Analytical Methods

All analyses were completed in RStudio Version 2024.12.0 + 467.

#### 3.4.1 Descriptive Statistics

We collected means, standard deviations, medians, and ranges of cognitive load metrics, patient characteristics during their encounter with the provider, and provider sex. We used the interquartile range (IQR) method to identify potential outliers in measures of EHR interaction complexity, including the number of loops, number of distinct events, total duration, and average time per event. Following the Tukey IQR rule, [28] values falling more than 1.5 times the IQR below the first quartile or above the third quartile were classified as outliers. A record was flagged as an outlier if any of the cognitive load metrics exceeded the Tukey thresholds.

#### 3.4.2 Associations Between Statin Prescribing and Cognitive Load Metrics

The primary outcome of interest is the likelihood of a cognitive load metric influencing a provider’s decision to initiate a statin for a patient. To do so, we ran a logistic regression analysis, with the dependent variable being whether a provider had initiated a statin for a patient who was eligible for statin therapy during an encounter in 2024. The independent variables of interest were each of the four cognitive load metrics: number of loops, average time spent on an EHR event, number of distinct EHR events, and duration of an encounter (Table 3). The controlled patient-level covariates included the patient’s sex, self-identified race and ethnicity, insurance status (aggregated as commercial or non-commercial), Elixhauser Comorbidity Index [29] during their visit, the number of active diagnoses during their encounter, and age. We included provider fixed effects to account for unobserved, time-invariant characteristics of each provider that may influence initiation decisions (e.g., years of active practice and number of other patients with similar conditions). In this healthcare system, physicians can see patients independently; however, NPs and PAs, despite having prescribing authority, must have a physician involved in the encounter. To adjust for multiple providers with prescribing abilities seeing a patient in the same encounter, we included clustered standard errors by the encounter ID. This combined approach helps isolate the effect of interest while producing conservative and robust standard errors.

However, cognitive load may have a nonlinear relationship with the probability of initiating a statin [30–32]. In some cases, clinical decision-making improves as cognitive challenge or task engagement increases, up to a saturation point, after which it declines, suggesting an optimal “middle ground” of effort. In other cases, low-effort task sequences may reflect automation of desired behaviors, whereas high-effort sequences may signal increased deliberation. In contrast, certain intermediate levels of effort could lead to inertia, resulting in no action being taken. Therefore, we introduced quadratic terms of each cognitive load metric to capture potential threshold effects flexibly. If the quadratic term did not statistically contribute to statin initiation, we tested whether excluding it would significantly impact the model fit by comparing the adjusted R^2^. If excluding the nonlinear term did not significantly impact the model fit, the quadratic term was removed to reduce the chance of overfitting the model. We calculated the average marginal effect of the metric on the likelihood of statin initiation for the cognitive load metrics with nonlinear terms to understand how much the likelihood of statin initiation changes for a particular amount of cognitive load. This approach enabled us to quantify both the direction and magnitude of the relationship across different levels of cognitive load, identifying points at which additional effort began to hinder rather than support statin initiation.

#### 3.4.3 Exploratory Analyses – Sources of Cognitive Load within the EHR

We aimed to link cognitive load measures within the EHR to statin initiation to identify opportunities for integrating clinical decision support. Using machine learning, we identified which EHR events were most strongly associated with statin initiation and the types of cognitive load associated with those events. We structured the EHR metadata so that each variable represented a cognitive load metric for a specific EHR event. This means, for example, we measured the average time a provider spent at Event A, how many times a provider looped back to Event B, and whether the provider visited Event C within an encounter. Because not every provider interacts with every EHR event, many of these variables contain zero values, reflecting events that were skipped or that did not have the cognitive load metric. To explore how these detailed interaction patterns relate to statin initiation, we used a gradient-boosted forest model that took these variables as inputs to predict whether a statin was initiated during the encounter. This machine learning method is well-suited for handling large, complex datasets with many variables, especially when relationships may be nonlinear (as per the earlier investigation) or involve interactions between events. It allowed us to identify which specific EHR events’ cognitive load metrics were most strongly associated with the decision to prescribe a statin. We tuned the forest to a learning rate (*η*) of 0.1 and a train-validation-test split of 70%−15%−15%. The procedure was repeated 200 times to generate a distribution of performance metrics, from which 95% confidence intervals were derived, offering a reliable assessment of the model’s performance and variability.

To interpret the model outputs, we applied Shapley Additive explanations (SHAP) values, which provide a theoretically grounded approach to attributing feature contributions to individual predictions. SHAP values offer both local interpretability, revealing how specific features influence each decision, and global interpretability, summarizing the overall importance of features across the dataset. [33] Mean SHAP values for each cognitive load-metric covariate and global mean SHAP values were computed to quantify the importance of each feature within the model in terms of direction (negatively or positively contributing to the prediction) and magnitude (how much the metric at the event contributes to overall prediction). Isolating the most significant contributors to the desired clinical behavior (i.e., statin initiation) with quantified contributions enables the future justification of introducing clinical decision support in the EHR.

## 4 Results

### 4.1 Descriptive Results

We analyzed 20,376 patient encounters across the academic healthcare system. The mean age of patients eligible for statin initiation was 58.8 years (SD = 10.8), and about half were male. The cohort was predominantly non-Hispanic/Latinx White, and approximately 80% had commercial insurance. Each patient had an average of 6 active diagnoses during their primary care provider encounter. Most encounters were handled by physicians (82.41%), with fewer by nurse practitioners (NP, 11.66%) and physician assistants (PAs, 5.93%). Encounters with NPs had the highest statin initiation rate (50.72% of NP encounters resulted in a statin initiation) compared to 46.86% for physicians and 46.6% for PAs. Encounters with statin initiation showed slightly lower cognitive load metrics on average than encounters with no statin. These differences were small but statistically significant: encounters with initiation had fewer loops, fewer distinct events, shorter duration, and a lower average time per event.

Overall, 17.6% of records had at least one extreme value in the cognitive load metrics (flagged as outliers per the Tukey IQR rule). A chi-squared test showed no significant difference in statin initiation rates between encounters with vs. without such outlier metrics (p = 0.20) – 45.7% vs 44.5% initiation, respectively. Risk analysis revealed that outlier encounters were 3% less likely to involve statin initiation (RR = 0.97; OR = 0.95, 95% CI 0.89–1.03), with no significant difference in the likelihood of statin prescribing between the two groups. In other words, extreme cognitive load metrics do not make an encounter more or less likely to result in a prescription. Therefore, we retained all observations in subsequent analyses. All descriptive analyses are presented in Table 4.

### 4.2 Regression Analyses between Cognitive Load Metrics and Statin Initiation

We examined whether the cognitive load metrics were associated with the likelihood of statin initiation in a multivariate logistic regression model, adjusting for patient characteristics and provider effects. Initially, we tested a model with only linear terms for each cognitive load metric; however, two of the cognitive load metrics—encounter duration (in minutes) and the average time spent per EHR event (in seconds)—did not show a significant linear relationship with statin initiation. We then examined non-linear relationships by adding quadratic terms. Model comparison revealed that quadratic terms for the number of loops and the number of distinct events significantly improved the fit (Wald test, p < 0.001). Thus, our final model included non-linear (quadratic) terms for loops and distinct events, as well as linear terms for duration and time per event. Comparisons of the linear and nonlinear models are presented in Table A.3.

#### 4.2.1 Linear Effect of Cognitive Metrics on Statin Initiation

Regression results are presented in Table 5. In the final regression model, the average time spent per EHR event and total encounter duration showed significant linear relationships with the initiation of statins. The average time spent per event showed a significant negative association with the likelihood of statin initiation, where spending more time per event was associated with a lower likelihood of initiation (β = −5.017 × 10^−4^, p < 0.001). On the other hand, the total duration of the encounter showed a significant positive association with the odds of initiating a statin, where longer encounter duration was associated with increased odds (β = 8.223 × 10^−3^, p < 0.001). These findings may reflect provider time management strategies, where extended engagement across multiple sections of the EHR is more conducive to initiating guideline-recommended treatment.

#### 4.2.2 Nonlinear Effects of Cognitive Metrics on Statin Initiation

**Figure 1:**
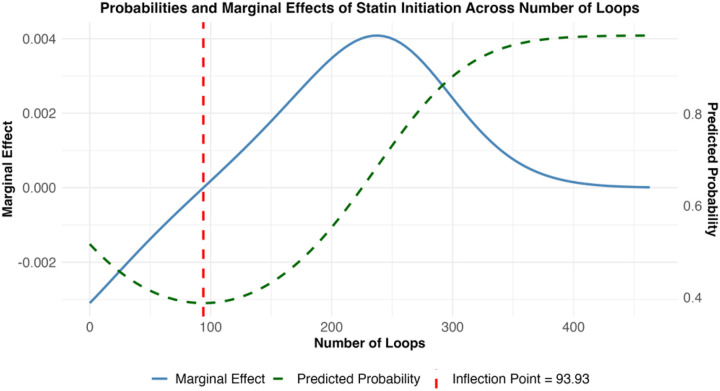
Plotted predicted probabilities and marginal effects of initiating a statin relative to the number of EHR event loops

**Figure 2:**
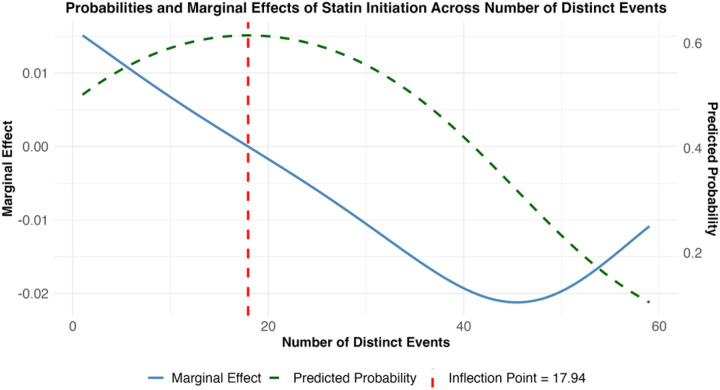
Plotted predicted probabilities and marginal effects of initiating a statin relative to the number of distinct EHR events

Figure 1 and Fig. 2 illustrate the two cognitive load metrics which exhibited significant nonlinear associations with statin initiation. For the number of loops, coefficients indicated a U-shaped relationship (linear β = −0.0124, p < 0.001; quadratic β = 0.597×10^−5^, p < 0.001). Encounters with either very low or very high loop counts were more likely to result in initiation than those with intermediate counts (Fig. 1, dashed green line). The inflection point occurred at 93.9 loops (Fig. 1, vertical dashed red line); below this threshold, each additional loop reduced the probability of initiation by 0.21%, whereas above it, each additional loop increased the probability by 0.00046%. The peak effect was observed at 237 loops, with a predicted initiation probability of 0.684.

The number of distinct events showed an inverse U-shaped relationship (linear model: β = 0.0124, p < 0.001; quadratic model: β = −0.597×10^−5^, p < 0.001), with the highest probability of initiation observed when encounters have moderate event counts. The inflection point occurred at approximately 18 events (Fig. 2, vertical dashed red line); below this threshold, each additional event increased the probability of initiation by 1.35%, whereas above it, the effect was negligible, and the probability gradually declined. This suggests an optimal range of distinct EHR actions that may facilitate decision-making regarding guideline-concordant statin initiation, with diminishing returns once complexity exceeds this threshold.

Patient race and sex were also significant predictors of statin initiation, as shown in Table 5. Compared to non-Hispanic/Latinx White patients, patients of racial and ethnic minorities were more likely to be initiated on a statin, as well as older patients. Female patients, as well as those with a greater number of active diagnoses or higher comorbidity burden, were less likely to receive initiation.

### 4.3 Exploratory Analyses for EHR Events Associated with Cognitive Load Metrics

We analyzed the EHR metadata with a machine learning model to pinpoint which specific EHR activities and associated cognitive load metrics were most strongly linked to statin initiation. We trained a gradient-boosted decision tree model. We computed SHAP values to quantify the contribution of each EHR interaction feature to the model’s predictions. The model achieved an area under the receiver operating characteristic curve (AUROC) of 0.91, indicating that using cognitive load metrics has strong performance in predicting statin initiation. Table 6 presents the 10 EHR event-specific features that contributed most to the model’s prediction, ordered by their global mean SHAP values and importance. A full list of contributions by cognitive load-EHR events can be found in Table A.4.

The average time per event (in seconds) had the highest relative contribution to predicting statin initiation, accounting for 72.2% of the model’s relative contribution to its predictions. There was a positive average SHAP value contribution, indicating a positive association between average time spent per event and statin initiation The number of loops accounted for 15.1% of the relative contribution but had a small negative average SHAP value contribution, indicating a negative association between loops and statin initiation. The presence of specific EHR events contributed 12.6% to the model’s predictions and had a small positive average contribution, indicating a positive association between the presence of an event and statin initiation.

The average time spent on viewing patient communications had a positive mean SHAP value of 0.0874, indicating that spending more time on a patient’s results was associated with a higher probability of statin initiation. This feature accounted for 23.1% of the model’s relative contribution to its predictions, making it the most influential predictor. The average time spent on suggested medication order sets had a positive mean SHAP value of 0.0129, indicating that spending more time on suggested medication order sets was associated with a higher probability of statin initiation. This feature accounted for 12.7% of the model’s relative contribution to its predictions, making it the second most influential predictor. The presence of the order set for testing contributed positively to predicting statin initiation, accounting for a 6.5% increase in the model’s predictive accuracy. Conversely, the average time spent on changing the list of orders showed a negative mean SHAP value of −0.0115, implying that modifying the order list was associated with a decreased likelihood of initiation, contributing approximately 7.74% to the model’s relative influence. Looping back to the order list contributed 3.74% to the model’s relative influence. It had a negative average SHAP value, indicating that the number of loops to change an order list was negatively associated with statin initiation.

These results suggest that interactions with the patient’s lab results and spending time on medication order sets positively influence prescribing behavior, while spending time on order modifications and looping back to change the orders may signal complexity or uncertainty, which reduces the likelihood of initiation.

## 5 Discussion

### 5.1 Summary

We identified statistically significant relationships between cognitive load metrics and the likelihood of initiating statin therapy during primary care encounters. First, we identified that cognitive load does not have a linear relationship to desired clinical behavior. Some types of cognitive load (in this paper’s case, engagement via repetitive behaviors) suggest that a “middle ground” of effort by providers increases the likelihood of a clinical behavior, that some effort, but not too much or too little effort, was associated with more appropriate prescribing. We also found that under-engagement (involving too few interactions) and over-engagement (characterized by excessive interactions) can positively impact clinical decision outcomes in statin initiation, possibly suggesting that either more active engagement or short, goal-directed engagement with patients leads to more appropriate prescribing behavior. These results highlight the dynamic nature of cognitive load in clinical environments. By applying machine learning techniques to EHR metadata, we identified key behaviors that current CDSS can leverage to support providers in delivering appropriate clinical care, as well as behaviors where existing EHR features may inadvertently hinder such decisions. It has been well documented that EHR nudges can result in alert fatigue; however, our work suggests that aptly designed CDS can optimize intended effect while reducing cognitive load. Specifically, CDS can create changes to interfaces when providers interact with a patient’s lab results, order medications, and work with alerts in the EHR that facilitate target actions. These findings underscore the potential of CDSS to enhance clinical decision-making and improve patient outcomes. [34]

### 5.2 Implications

This study provides actionable insights into how cognitive load in EHR workflows affects clinical decision-making behavior and presents a framework for how to improve clinical decision-making. By measuring cognitive load indicators within the EHR and identifying specific interaction patterns associated with prescribing decisions, we highlight key moments in the workflow that can be targeted with more effective CDS tools. These findings lay the groundwork for designing interventions that reduce unnecessary complexity and better support clinicians in following guideline-recommended therapies.

Our methodological approach provides both immediate and long-term benefits for health systems. In the short term, these insights can help health systems identify workflow gaps that hinder the provision of effective decision support. Over the long term, this approach has the potential to transform how health systems monitor and improve provider behavior, enabling data-driven quality initiatives. These methods can be further refined to distinguish between helpful and unhelpful cognitive load for providers, ultimately leading to better patient care through intentional, thoughtful digital actions. For patients, improved prescribing practices can have a direct and meaningful impact on outcomes, particularly by increasing the use of appropriate statins to lower primary ASCVD risk.

The developed methodology is scalable and adaptable to various clinical settings and EHR systems. Our data processing and workflow characterization techniques can support a wide range of efforts to enhance clinical decision support and promote evidence-based care at scale. Firstly, our approach to extracting audit log metadata and clinical behavior data, and linking these to clinical data, can be easily adapted for other behavior-related questions, such as specialty referrals, shared decision-making, and screening choices. Secondly, the identification of behaviors within the EHR and the creation of cognitive load metrics are independent of any specific EHR, as they rely on process mining and data science techniques. The analytical methods used to examine the relationship between cognitive load and clinical behavior, along with machine learning approaches that connect cognitive load to EHR events, offer a systematic way to evaluate CDSS designed to improve clinical practices and identify behaviors that need adjustment at specific interfaces.

### 5.3 Limitations

Several limitations should be considered when interpreting our findings. First, the cognitive load and workflow measures used in this study were intentionally simple. While more sophisticated process mining techniques, such as clustering workflow sequences or calculating transition probabilities between EHR events, could yield deeper insights, we prioritized interpretable, actionable metrics aligned with behavior change theory. These simpler measures are more readily translatable into tools within the EHR to support clinical decision-making. Moreover, the application of EHR metadata for healthcare quality improvement remains an emerging area within clinical informatics. There is a pressing need for standardization of EHR metadata metrics by organizations such as the Office of the National Coordinator for Health Information Technology (ONC), which could also enable validation of more complex measures through direct observation or qualitative research.

Second, our findings are based on data from a single EHR platform within a single academic health center’s EHR system. While the specific events captured may vary across institutions, our computational approach was designed to be generalizable by focusing on abstracted, system-agnostic event types. Thus, while implementation may require adaptation to local data structures, the methodology should be transferable to other EHR platforms.

Third, the cognitive load metrics used in this study do not distinguish between intrinsic, extrinsic, and germane cognitive load. As a result, we cannot determine whether the measured cognitive load metrics reflect the inherent complexity of the clinical decision (intrinsic load), inefficiencies or distractions introduced by the EHR interface or workflow (extrinsic load), or the cognitive effort invested in understanding and integrating information for decision-making (germane load). Without this differentiation, it is difficult to target design interventions precisely, like reducing extrinsic load without inadvertently removing beneficial germane load. Future work incorporating methods such as task analysis, think-aloud protocols, or real-time workload assessment could help disentangle these components and guide more tailored improvements to CDSS and EHR.

Fourth, our current methods do not link each EHR action to a specific medication, lab, or diagnosis. This allowed us to compare all encounters with and without statin initiation in a general way. In future work, we could focus on encounters that involve the same medications and labs and see how cognitive effort on each specific action—like reviewing cholesterol lab results or adjusting a statin order—affects whether a statin is prescribed.

Fifth, this study focused on one clinical use case: initiating maintenance medications (statins) in primary care. Although our population identification and data linkage methods were tailored to this context, the overall approach can be applied to other clinical domains. Importantly, the metadata metrics were derived independently of the clinical content, which supports broader applicability. With further development, EHR vendors and health systems could aggregate and standardize such metrics to inform workflow improvement and decision support across various clinical scenarios. For example, the scope of the study was intentionally limited to initiation and did not include decisions regarding the titration of statins. Further research can elaborate on differentiating the cognitive load of workflows for initiation versus titration, given the distinct behavioral mechanisms and decision-making processes associated with each. This study also limited its analysis to the behavior of providers, despite evidence of patient hesitancy to start statin therapy.[35] The methods of this study are equally applicable to the EHR audit log metadata of patient-facing interfaces and patient portals, to enhance the digital patient experience.

## 6 Conclusion

Our findings suggest that the likelihood of initiating statins is not simply determined by the amount of effort in the EHR but is influenced by a nonlinear relationship between cognitive load and decision-making. Both too little and too much engagement with the EHR can decrease the chances of initiating appropriate therapy, while an optimal level of engagement seems to promote guideline-concordant prescribing. Similarly, extreme engagement behaviors in the EHR can support clinical actions, whereas a moderate approach may lead to low engagement. By identifying specific EHR interaction patterns and workloads that either facilitate or hinder statin initiation, our results highlight practical opportunities to redesign clinical decision support and workflows to better balance cognitive demands. Efficient, targeted EHR use—rather than maximal or minimal effort—may be the most effective way to ensure timely and appropriate treatment decisions.

Importantly, our study addresses and overcomes significant barriers and challenges associated with using EHR metadata. We demonstrate that these complex metrics can be extracted, processed, and mapped to detailed clinical workflows, enabling robust analysis of how cognitive load impacts clinical decision-making. This advancement provides a framework for health systems to better understand and optimize EHR interactions, ultimately supporting improved guideline adherence and patient outcomes.

## Supplementary Material

Supplementary Files

This is a list of supplementary files associated with this preprint. Click to download.

- AuditLogManuscriptAppendixTables.xlsx

## Figures and Tables

**Table 2:** Inclusion and Exclusion Criteria for Statin Therapy Eligibility

Inclusion Criteria	Exclusion Criteria
Patients with any of the following guidelines: Age 20 to 75, with LDL ≥ 190 mg/dL and diagnosis of Type 2 Diabetes Mellitus^1^ Age 40 to 75, with Type 2 Diabetes Mellitus^1^ and a history of myocardial infarction (MI)^2^ or stroke^2^ Age 40 to 75, with an estimated 10-year ASCVD risk ≥ 7.5% Age 20 to 75, with a history of myocardial infarction (MI)^2^ or stroke^2^	Patients with any of the following conditions: Pregnant Have a known allergy to statins or a contraindication to statin use Have any of the following diagnoses: Hepatitis^2^ Cirrhosis^2^ End-Stage Renal Disease (ESRD)^2^ Chronic Kidney Disease (CKD)^2^

1 Type 2 Diabetes Mellitus identified via ICD-10 codes (Table A.2.) or HbA1c ≥ 6.5% in their latest blood draw.

2 Diagnoses identified using ICD-10 codes listed in Table A.2.

**Table 3:** Cognitive Load Metric Definitions

Cognitive Load Metric	Definition	Example of Cognitive Load Metric	Associations between Metric and Opportunity for Behavior Change
Number of Loops (N)	The number of times an event is revisited during an encounter.	User moves from Event A → Event B → Event C → Event A → Event B → Event D (2 loops total – one loop back to Event A and one loop back to Event B)	More loops could increase cognitive effort due to redundancy or confusion. More loops suggest redesigning workflows to remove unnecessary repetition.
Average Time Spent on an EHR Event (seconds)	The average time spent on an EHR event.	8.7 seconds per event (e.g., Event A, Event B, Event C)	More time spent per event indicates complexity and more fixating, suggesting opportunities for simplification or support.
Number of Distinct EHR Events	The number of unique events in the EHR that a provider visits during their encounter.	15 unique events (Event A, Event B, Event C, …, Event O)	More distinct events increase task complexity and intrinsic load. More distinct events suggest chunking related tasks or adding grouping mechanisms.
Duration of an Encounter (minutes)	The total active time an agent spends engaging with data or actions related to a specific customer interaction, summed across all non-contiguous time segments associated with the same encounter ID.	17.9 minutes from first event (A) to last event (C) related to an encounter	Longer encounter duration indicates longer time engaging with the EHR for reviewing patient data, which could introduce fatigue or signal focused patient engagement. Shorter encounter duration may be related to goal-oriented behaviors, resulting in decreased cognitive load.

**Table 4:** Descriptive statistics of patients, providers, and cognitive load metrics

	No Initiation	Initiation	P-value
	(N = 11105 Patients)	(N = 9271 Patients)	
**Patient Characteristics**			
**Age (years)**			
Mean (SD)	58.9 (10.8)	58.7 (10.8)	0.211
Median [Min, Max]	61.0 [20.0, 75.0]	60.0 [20.0, 75.0]	
**Sex**			
Male	5542 (49.9%)	4788 (51.6%)	0.0141
Female	5560 (50.1%)	4483 (48.4%)	
Unknown	3 (0.0%)	0 (0%)	
**Race Ethnicity**			
Non-Hispanic/Latinx White	6612 (59.5%)	4366 (47.1%)	<0.001
Non-Hispanic/Latinx Black/African American	1434 (12.9%)	1098 (11.8%)	
Hispanic/Latinx	1091 (9.8%)	1791 (19.3%)	
Asian	844 (7.6%)	723 (7.8%)	
Other	1124 (10.1%)	1293 (13.9%)	
**Financial Class**			
Commercial	8907 (80.2%)	7263 (78.3%)	0.00111
Non-commercial	2198 (19.8%)	2008 (21.7%)	
**Number of Active Diagnosis**			
Mean (SD)	6.40 (3.07)	6.09 (3.51)	<0.001
Median [Min, Max]	6.00 [1.00, 24.0]	5.00 [1.00, 32.0]	
**Elixhauser Comorbidity Index**			
Mean (SD)	1.68 (5.42)	1.42 (5.37)	<0.001
Median [Min, Max]	0 [−14.0, 42.0]	0 [−18.0, 58.0]	
**Number of Providers Involved in Encounter**			
One	8133 (73.2%)	6866 (74.1%)	
Two	2637 (23.7%)	2154 (23.2%)	
Three	335 (3.0%)	251 (2.7%)	
**Provider Type**	**Physician**	**Nurse Practitioner**	**Physician Assistant**
Number of Encounters	33281 (82.41%)	4709 (11.66%)	2393 (5.93%)
**Initiation Rate**			
Mean (SD)	46.86 (0.77)	50.72 (1.47)	46.60 (2.00)
Median [Min, Max]	46.15 [0, 100]	50.00 [0, 100]	41.05 [0, 100]
	**No Initiation**	**Initiation**	
**Cognitive Load Metrics**	**(N = 11105 Encounters)**	**(N = 9271 Encounters)**	**P-value**
**Number of Loops**			
**Patient Characteristics**			
Mean (SD)	75.0 (34.2)	71.1 (39.4)	<0.001
Median [Min, Max]	70.0 [1.00, 311]	63.0 [0, 465]	
**Number of Distinct Events**			
Mean (SD)	34.0 (6.01)	33.4 (7.00)	<0.001
Median [Min, Max]	34.0 [8.00, 69.0]	33.0 [9.00, 80.0]	
**Encounter Duration (Minutes)**			
Mean (SD)	38.8 (20.0)	37.8 (22.1)	<0.001
Median [Min, Max]	35.0 [1.17, 183]	33.0 [0.333, 246]	
**Average Time per EHR Event (Seconds)**			
Mean (SD)	44.5 (61.3)	40.9 (55.9)	<0.001
Median [Min, Max]	22.7 [1.84, 970]	22.0 [1.11, 791]	

**Table 5:** Coefficients of the nonlinear model associating cognitive load with statin initiation.

	Reference Level	Coefficient	Std. Err	Statistic
Number of Loops	--	−1.239e − 02***	(2.123e − 03)	−5.84
Number of Loops^2^		6.597e − 05***	(1.021e − 05)	6.46
Number of Distinct Events	--	6.412e − 02***	(8.381e − 03)	7.65
Distinct Events^2^		−1.787e − 03***	(1.762e − 04)	−10.1
Total Duration (min)	--	8.223e − 03***	(1.436e − 03)	5.73
Avg Time Per Event (sec)	--	−5.017e − 04***	(1.314e − 04)	−3.82
Race: Black/African American	Non-Hispanic/Latinx White	1.967e − 01**	(6.567e − 02)	3
Race: Hispanic/Latino	--	5.906e − 01***	(6.508e − 02)	9.08
Race: Asian	--	4.425e − 01***	(8.083e − 02)	5.47
Race: Other		6.839e − 01***	(6.363e − 02)	10.7
Patient Age		1.023e − 02***	(2.101e − 03)	4.87
Patient Sex: Female	Male	−8.218e − 02*	(4.103e − 02)	−2
Patient Sex: Unknown		−2.016e + 01***	(5.316e − 02)	−379
Insurance: Non-commercial	Commercial	5.738e − 02	(5.466e − 02)	1.05
Number of Active Diagnoses		−4.879e − 02***	(7.546e − 03)	−6.47
Elixhauser Comorbidity Index		−9.656e − 03**	(3.742e − 03)	−2.58
N Observations		36131		
R^2^		0.2		
R^2^ Adj.		0.128		
RMSE		0.43		

+ p < 0.1,

* p < 0.05,

** p < 0.01,

*** p < 0.001

**Table 6:** Top 10 cognitive load features associated with statin initiation, ranked by mean absolute SHAP value. The relative contribution of these features is 70.06% of the model’s predictions.

Rank	EHR Event	Cognitive Load Metric	Mean SHAP	Mean Absolute SHAP	95% CI Lower	95% CI Upper	Contribution Direction	Relative Contribution
**1**	Patient communications viewed	Average Time Per Event (sec)	0.0874	0.7205	0.1014	0.0734	Increasing	0.2313
**2**	Order set suggested	Average Time Per Event (sec)	0.0129	0.3967	0.0200	0.0059	Increasing	0.1274
**3**	Order modified	Average Time Per Event (sec)	−0.0115	0.2410	−0.0064	−0.0166	Decreasing	0.0774
**4**	Order set suggested	Event Shown (1/0)	0.0115	0.2030	0.0151	0.0078	Increasing	0.0652
**5**	Patient’s lab results reviewed	Average Time Per Event (sec)	0.0293	0.1321	0.0322	0.0264	Increasing	0.0424
**6**	Order list changed	Number of Loops	−0.0090	0.1164	−0.0068	−0.0112	Decreasing	0.0374
**7**	Order set activity selected	Average Time Per Event (sec)	0.0043	0.1091	0.0066	0.0019	Increasing	0.0350
**8**	Alert displayed	Average Time Per Event (sec)	−0.0051	0.1004	−0.0034	−0.0069	Decreasing	0.0322
**9**	Problem List modified	Number of Loops	−0.0021	0.0826	−0.0002	−0.0041	Decreasing	0.0265
**10**	Chart Review Encounter report viewed	Average Time Per Event (sec)	−0.0024	0.0807	−0.0010	−0.0038	Decreasing	0.0259

## Data Availability

Due to the use of patient data in this study, deidentified versions of the data can be made available upon reasonable request. Dr. Safiya Richardson can be contacted at Safiya.richardson@nyulangone.org for data requests.

## Abbreviations

Atherosclerotic cardiovasculardisease (ASCVD): Condition caused by plaque buildup (atherosclerosis) in the arteries
ASCVD 10-year risk score: Estimate of a person’s chance of having a heart attack or stroke within the next 10 years
Clinical Decision Support Systems(CDSS): Tools or systems within the EHR that provide clinicians with information, alerts, or recommendations to improve patient care decisions.
Cognitive Load: The mental effort required to process information and complete tasks, influenced by the complexity of the information, the environment, and the interface design.
Electronic Health Record (EHR): A digital system for storing and managing patient medical records and clinical workflows.
EHR Metadata: Data automatically generated and stored by the EHR that describes user interactions, such as keystrokes, clicks, screen time, and navigation patterns.
Gradient-boosted forests: Machine learning method that combines decision trees built in sequence in which new trees correct errors of previous trees to improve accuracy
Hadoop distributed file system: System for storing large data sets across multiple machines, allowing processing in parallel
Hawthorne Effect: When people change their behavior because they are aware they are being observed
AUC-ROC (Area Under the Curve – Receiver Operating Characteristic): A measure of a binary classifier’s performance; higher values indicate better discrimination between positive and negative cases.
Random Forest: A machine learning method that uses multiple decision trees to make predictions or classifications; in this study, used to predict statin initiation from cognitive load metrics.
Shapley additive explanation(SHAP) values: Method to explain machine learning model’s predictions by quantifying each feature’s contribution to a specific prediction
Statin: A class of cholesterol-lowering medications used to reduce cardiovascular risk, often prescribed for patients with elevated ASCVD risk, by blocking the production of low-density lipids (LDL) in the liver, helping prevent heart attacks and strokes.
STROBE: The Strengthening the Reporting of Observational Studies in Epidemiology (STROBE) guidelines for reporting observational studies
Tukey IQR rule: A statistical method for identifying outliers by defining data points lying more than 1.5 times the interquartile range above the third quartile or below the first quartile.
